# CORM-3 Attenuates Oxidative Stress-Induced Bone Loss via the Nrf2/HO-1 Pathway

**DOI:** 10.1155/2022/5098358

**Published:** 2022-08-17

**Authors:** Chen Jin, Bing-hao Lin, Gang Zheng, Kai Tan, Guang-yao Liu, Zhe Yao, Jun Xie, Wei-kai Chen, Liang Chen, Tian-hao Xu, Cheng-bin Huang, Zong-yi Wu, Lei Yang

**Affiliations:** ^1^Department of Orthopedic, The Second Affiliated Hospital and Yuying Children's Hospital of Wenzhou Medical University, Wenzhou 325000, China; ^2^Key Laboratory of Orthopedics of Zhejiang Province, Wenzhou 325000, China; ^3^Department of Burn and Wound Center, The First Affiliated Hospital of Wenzhou Medical University, Wenzhou 325000, China; ^4^School of Medicine, Shanghai University, Shanghai 200444, China; ^5^Orthopaedic Oncology Services, Department of Orthopedics, The Second Affiliated Hospital of Zhejiang University School of Medicine, Hangzhou 310009, China

## Abstract

Bone metabolism occurs in the entire life of an individual and is required for maintaining skeletal homeostasis. The imbalance between osteogenesis and osteoclastogenesis eventually leads to osteoporosis. Oxidative stress is considered a major cause of bone homeostasis disorder, and relieving excessive oxidative stress in bone mesenchymal stem cells (BMSCs) is a potential treatment strategy for osteoporosis. Carbon monoxide releasing molecule-3 (CORM-3), the classical donor of carbon monoxide (CO), possesses antioxidation, antiapoptosis, and anti-inflammatory properties. In our study, we found that CORM-3 could reduce reactive oxygen species (ROS) accumulation and prevent mitochondrial dysfunction thereby restoring the osteogenic potential of the BMSCs disrupted by hydrogen peroxide (H_2_O_2_) exposure. The action of CORM-3 was preliminarily considered the consequence of Nrf2/HO-1 axis activation. In addition, CORM-3 inhibited osteoclast formation in mouse primary bone marrow monocytes (BMMs) by inhibiting H_2_O_2_-induced polarization of M1 macrophages and endowing macrophages with M2 polarizating ability. Rat models further demonstrated that CORM-3 treatment could restore bone mass and enhance the expression of Nrf2 and osteogenic markers in the distal femurs. In summary, CORM-3 is a potential therapeutic agent for the treatment of osteoporosis.

## 1. Introduction

Osteoporosis is one of the most common metabolic bone diseases worldwide, characterized by bone mass reduction and bone microarchitecture deterioration. This weakens the bones, making them fragile and prone to fracture. The related treatment costs and weak human resources present substantial clinical and economic impacts [1–3]. The pathogenesis of osteoporosis is caused by numerous factors, with oxidative stress being central [4, 5]. Hydrogen peroxide and superoxide ions are thought to interfere with the bone environment in two main ways: suppressing osteoblastic functions and increasing osteoclastic activity [6, 7]. Hence, modulating ROS production is a potential strategy for osteoporosis therapies [8].

Heme oxygenase-1 (HO-1) is a highly conserved enzyme that degrades heme into biliverdin, CO, and Fe^2+^ [9, 10]. Gierdanella et al. reported that HO-1 is significantly downregulated in diabetic retinopathy [11]. Similarly, Chen et al. demonstrated that overexpression of the HO-1 gene attenuates myocardial infarction and ventricular arrhythmias after myocardial ischemia and reperfusion in rats [12]. Recently, mouse models revealed that HO-1 expression is downregulated considerably in ovariectomy (OVX) [13]. A low concentration of CO, a catalytic product of HO-1, regulates various biological activities, including inflammation, apoptosis, and oxidation, which explains the protective effect of HO-1 [14, 15].

CORM-3, a safe and readily available alternative to CO, has been widely used to investigate the working mechanisms of living systems [16, 17]. Also, it can be used in treating several diseases. Recently, CORM-3 has been reported to ameliorate spinal cord-blood barrier disruption following injury to the spinal cord and alleviate neuron death after spinal cord injury via inflammasome regulation by Zheng et al. [18]. Also, it has been proposed that CORM-3 promotes the osteogenic differentiation of BMSCs and enhances the osteogenic differentiation of human periodontal ligament stem cells [19, 20]. However, how CORM-3 ameliorates oxidative stress-induced damage is unclear. In the present study, oxidative stress was induced in BMSCs using H_2_O_2_ as previously reported [21].

Therefore, we first measured the changes in CO concentration and the expression of HO-1 in normal and OVX rats. We found that in the OVX rats, the changes in CO contents correlated with HO-1 expression after OVX. Furthermore, we found that exogenously increasing CO by CORM-3 treatment rescued their osteogenic differentiation capacity and reduced apoptosis in the ROS environment. In addition, the injection of CORM-3 restored the bone microstructure and protected ovariectomized rats against bone loss in vivo. This study showed the mechanism and potential application of CO for osteoporosis therapy.

## 2. Materials and Methods

### 2.1. Antibodies, Reagents, and Media

CORM-3 and H_2_O_2_ were purchased from Sigma-Aldrich (St. Louis, MO, USA). Anti-Collagen Type I Alpha 1 (COL1A1), Runt-related transcription factor 2 (RUNX2), and osteocalcin (OCN) antibodies were purchased from Cell Signalling Technology (Denver, MA, USA). Cytochrome C (Cyto-C), Bax, cleaved-caspase3, and Bcl-2 were purchased from Proteintech (Chicago, IL), whereas nuclear factor erythroid 2-related factor 2 (Nrf2), HO-1, NAD(P) H:quinone oxidoreductase 1 (NQO1), *β*-actin, Lamin B, and GAPDH antibodies were purchased from ABCom (Cambridge, MA). MEM Alpha Modification (*α*-MEM), fetal bovine serum (FBS), and penicillin/streptomycin were purchased from Life Technologies (Rockville, MD). All reagents used in this study were of analytical grade or met the criteria required for cell culture experiments.

### 2.2. Isolation and BMSC Culture

BMSCs extracted from the femur of Sprague-Dawley rats were cultured as previously described [22]. Briefly, BMSCs were isolated from the femur bone of euthanized Sprague-Dawley rats, sterilized for 15 minutes in 70% ethanol, and rinsed three times with phosphate-buffered saline (PBS). The BMSCs were then suspended in *α*-MEM supplemented with 10% (*v*/*v*) FBS and 104 IU/mL penicillin/streptomycin. Nonadherent BMSCs after 12 hours of incubation were removed, whereas the adherent cells were cultured in fresh media for follow-up experiments, which were changed every two to three days.

### 2.3. Cell Proliferation and Apoptosis Assay

The effects of CORM-3 and H_2_O_2_ on the viability of BMSCs were assessed using the Cell Counting Kit 8 (CCK-8) kit (Beyotime Institute of Biotechnology). The cells were inoculated in 96-well plates at a density of 5 × 10^3^and treated for 2 hours with varying CORM-3/iCORM concentrations (0, 12.5, 25, 50, 100, 200, and 400 *μ*m) with or without H_2_O_2_ treatment. After 24 hours, 10 *μ*L CCK-8 reagent was added to each well before further culturing for another 1 hour. The optical density in each was measured at 450 nm using a spectrophotometer (Thermo Fisher Science, Waltham, MA, USA).

The proliferation of cells was performed using EDU Cell Proliferation Kit (C0071L, Beyotime, China) according to manufacturer's instructions [23]. Briefly, BMSCs were incubated for 4 hours with EDU and fixed for 30 minutes using 4% polyformaldehyde. Nuclear staining was performed using Hoechst. The stained cells were observed and photographed using a microscope.

The effect of CORM-3 and H_2_O_2_ on the apoptosis of BMSCs was assessed using the FITC Annexin V/PI Apoptosis Detection Kit (Beyotime Institute of Biotechnology). Briefly, 1 × 10^5^ cells were washed twice using cold PBS and resuspended in 1× binding buffer. The cells were then stained using propidium iodide and Annexin V FITC. Flow cytometry was used to detect early and late cellular apoptosis. The data were analyzed using the FlowJo software.

### 2.4. Osteogenic Differentiation of BMSCs

BMSCs were first seeded into 24-well plates for osteogenic differentiation at an initial density of 5 × 10^4^ cells/well. After treatment, the culture medium was replaced with an osteogenic medium. The medium was changed every day. Alkaline phosphatase (ALP) and Alizarin red S (ARS) staining were performed as previously described [24]. Staining was performed on osteogenic days 7 and 21 using the ALP Staining Kit (Beyotime Institute of Biotechnology; Jiangsu, China) and the ARS solution (Solarbio Science & Technology). The absorbance of ALP and ARS was measured using a microplate reader at 520 nm and 570 nm, respectively.

### 2.5. Carbon Monoxide Content Detection in Femur

The CO content in different groups of rats was detected using the Endogenous Carbon Monoxide Assay Kit (Jiancheng Biotechnology, Nanjing, China) as previously described [25]. Briefly, the distal femur bones for rats in both groups (sham group and OVX group) were cut into small pieces and washed in PBS. After homogenizing in PBS, 0.5 mL of the tissues were added into the Hb solution (1 mL). Following vortexing and quiescence, mixtures were measured at 541 nm (as absorbance) and 555 nm (as a reference). The CO content in samples was based on the ratio of *x* and *y* measured at 541 nm and 555 nm.

### 2.6. Western Blot Analysis

Western blot analysis was performed as previously described [26]. BMSCs were washed for 30 minutes at 4°C with ice-cold PBS and lysed with radioimmunoprecipitation (RIPA) lysis buffer (Beyotime Institute of Biotechnology) supplemented with phosphatase and protease inhibitors (1 mM) (Sigma-Aldrich). The cell lysate was centrifuged at 12000 g to separate the proteins. The concentration of the proteins was evaluated using the BCA Protein Assay Kit (Beyotime Institute of Biotechnology). SDS–polyacrylamide gel electrophoresis (SDS-PAGE) was performed using 20 *μ*g of the extracted proteins. Briefly, the proteins were first transferred to a polyvinylidene difluoride membrane through overnight incubation at 4°C. After three washes with 0.1% Tween 20 (TBST), the membranes were incubated for 4 hours at room temperature with corresponding HRP-conjugated secondary antibodies. The proteins were visualized using a ChemiDoc XRS+ system equipped with an augmented chemiluminescence reagent (Bio-Rad; Hercules, CA, USA). The various protein bands were quantitatively analyzed using the Image Lab software V3.0 (Bio-Rad).

### 2.7. Quantification of the Activities of CAT and GSH

After treatment with H_2_O_2_, the cells were rinsed three times with PBS and lysed for 30 minutes on ice-cold lysis buffer. The activities of CAT and GSH were assessed using commercial assay kits (Jiancheng Biotechnology, Nanjing, China).

### 2.8. Lentivirus Transfection

Nrf2 knockdown in BMSCs was performed through transfection with lentivirus (GeneChem, Shanghai, China) at a density of 30-50% density. The culture media of the cells was replaced after twelve hours after transfection and replaced every three days replaced until the growth of cells reached 80-90% confluency. The efficacy of transfection was analyzed using western blot.

### 2.9. Reactive Oxygen Species Assay

The levels of intracellular ROS were quantified using the dihydroethidium (DHE) test (Yeasen Biotech Co., Ltd., Shanghai, China) based on a fluorescent probe. Treated BMSCs were washed three times using PBS buffer and incubated at 37°C for 30 minutes in darkness with DHE. The cells were observed immediately under a microscope (Olympus Life Science; Tokyo, Japan). Three random photos were then captured and analyzed using the Image-Pro Plus software version 6.0.

### 2.10. Mitochondrial Function Assays

The levels of superoxide ions and the mitochondrial membrane potential (MMP) in treated BMSCs were determined by MitoSox and JC-1 probes, respectively, according to the manufacturer's instructions (Beyotime, Shanghai, China). The cells were observed using a fluorescent microscope (Olympus Life Science; Tokyo, Japan).

### 2.11. RNA Extraction and Real-Time Quantitative Polymerase Chain Reaction

The gene expression levels in polarized macrophages were assessed using real-time quantitative polymerase chain reaction (qPCR). Total RNA was extracted from cultured cells using TRIzol (Invitrogen, Carlsbad, CA, USA). Then, 1 *μ*g of RNA was transcribed to cDNA using oligo dT primers before amplifying 2 *μ*g of each cDNA. The primer sequences used in this study included the following: 5′-GGAGTGACGGCAAACATGACT-3′ (sense) and 5′-TCGATGCACAACTGGGTGAAC-3′ (antisense) for inducible nitric oxide synthase (iNOS); 5′-CTGAACTTCGGGGTGATCGG-3′ (sense) and 5′-GGCTTGTCACTCGAATTTTGAGA-3′ (antisense) for tumor necrosis factor-*α* (TNF-*α*); 5′-GAAATGCCACCTTTTGACAGTG-3′ (sense) and 5′-TGGATGCTCTCATCAGGACAG-3′ (antisense) for interleukin-1*β* (IL-1*β*); 5′-TTGGGTGGATGCTCACACTG-3′ (sense) and 5′-GTACACGATGTCTTTGGCAGA-3′ (antisense) for arginase-1 (Arg-1); 5′-CAGGTCTGGCAATTCTTCTGAA-3′ (sense) and 5′-GTCTTGCTCATGTGTGTAAGTGA-3′ (antisense) for chitinase-like 3 (Chil3/Ym-1); 5′-GCTTCCGTGGTTCCATAGACC-3′ (sense) and 5′-TCATCCGTGGTTCCATAGACC-3′ (antisense) for macrophage mannose receptor (CD206); and 5′-AAGAAGGTGGTGAAGCAGG-3′ (sense) and 5′-GAAGGTGGAAGAGTGGGACT-3′ (antisense) for GAPDH.

### 2.12. Tartrate-Resistant Acid Phosphatase (TRAP) Activity and Staining

Briefly, BMMs were seeded into wells of 96-well plates at the density of 8 × 10^3^ cells/per well. After 24 hours, the cells were treated with receptor activator of nuclear factor-kappa B ligand (RANKL) (50 ng/mL), macrophage colony-stimulating factor (M-CSF) (30 ng/mL), H_2_O_2_ (20 *μ*m), and CORM-3 (100 *μ*m). The culture media was replaced every two days until the BMMs developed into mature osteoclasts. The cells were fixed for 20 minutes with 4% paraformaldehyde. Osteoclasts were TRAP-positive cells (with ≥3 nuclei/cell) as seen under a light microscope.

### 2.13. Transmission Electron Microscopy (TEM)

First, the treated BMSCs were first fixed at 4°C for 12 hours with 2.5% glutaraldehyde and then stained negatively for 1 hour at room temperature with 2% uranyl acetate. The temperature of the cells was raised gradually to room temperature before washing the cells with acetone. The cells were then embedded in epoxy resin, grouped, and stained with toluidine blue. Then, the cells were then observed under the Hitachi Field Emission Transmission Electron Microscope (Hitachi High-Technologies Corp; Tokyo, Japan).

### 2.14. Immunofluorescence

BMSCs were cultured on glass slides at 37°C for 20 minutes and permeabilized with 0.1% (*v*/*v*) Triton X-100 in PBS for 15 minutes. Then, the cells were incubated for 1 hour at room temperature with 1% goat serum to block nonspecific antibody binding sites. The cells were then incubated at 4°C overnight with primary antibodies rinsed three times with PBS and further incubated at room temperature for 1 hour in the dark with fluorescence-conjugated secondary antibodies. After staining the cell nuclei with DAPI for 5 minutes at room temperature, the cells were visualized using an Olympus fluorescent microscope (Olympus Life Science; Tokyo, Japan). The IF results were analyzed quantitatively using the Image-Pro Plus 2D Software (Rockville, MD, USA).

### 2.15. Treatment and Animal Model

The protocol for animal experiments was approved by the Ethics Committee of the Second Affiliated Hospital and Yuying Children's Hospital of Wenzhou Medical University. A total of 40 female SD rats (three months of age and weighed 250 ± 20 g) used in this study were purchased from Shanghai Laboratory Animal Center (SLACCAS; Shanghai, China) and were maintained under specific pathogen-free (SPF) conditions at 22-25°C under a 12-hour light/dark cycle. The rats were provided with enough food and water. Inactive CORM-3 (iCORM-3) was generated by overnight incubation of CORM-3 in PBS at room temperature to assess the effect of CO. The rats were divided into four groups. The sham group consisted of ten randomly picked rats. The remaining 30 rats underwent bilateral OVX using the double dorso-lateral method described by Park et al. OVX rats were randomly divided into OVX+CORM-3, OVX+iCORM-3, and OVX+saline groups (OVX group). The OVX+CORM-3 group received 15 mg/kg of CORM-3 diluted with normal saline for 8 weeks. At the same time, the OVX group and OVX+iCORM-3 group received an intraperitoneal injection of an equivalent volume of saline and 15 mg/kg of iCORM-3, respectively, for 8 weeks. The femur bones of the rats were collected and preserved in 4% paraformaldehyde after sacrificing the rats.

### 2.16. The Microstructure of the Distal Femur

The microstructure of the distal femur was analyzed using a micro-CT imaging system (*μ*CT 100, Scanco Medical; Bruttisellen, Switzerland). The microarchitectural parameters of the volume of interest (VOI) were obtained using software in the micro-CT workstation, based on three-dimensional reconstructed images. Finally, further bone quality assessment was performed by quantitatively measuring region of interest- (ROI-) related parameters, including the bone volume to tissue volume (BV/TV), trabecular thickness (Tb.Th, mm), trabecular number (Tb.N, 1/mm), and trabecular separation (Tb.Sp, mm).

### 2.17. Calcein Labeling Assay

Rats were injected intraperitoneally with 20 mg/kg calcein (Sigma-Aldrich, USA) on days 14 and 2 before being sacrificed. After sacrifice, the left femur bone was removed and fixed in 4% formaldehyde and embedded in methyl methacrylate. After hardening, the bone tissue was dissected into 30 *μ*m thin sections using a microtome (SP1600; Leica, Germany). The cortical endosteum surfaces were evaluated immediately using a fluorescence microscope (STP6000; Leica, Germany) with an excitation wavelength of 488 nm.

### 2.18. Histomorphological and Immunohistochemical (IHC) Analyses

After CT scanning, the femur bones were decalcified for 3 weeks in 10% EDTA solution, embedded in paraffin wax, fixed in ethanol, cleared in xylene, and embedded in paraffin wax. The bones were then cut longitudinally into 4 *μ*m thick sections and mounted on polylysine-coated glass slides before hematoxylin and eosin (H&E) and Masson's trichrome staining (Solarbio Science & Technology). For IHC analysis, 4 *μ*m thick sections were incubated with primary antibodies against Nrf2, COL1A1, HO-1, and RUNX2, rinsed three times using PBS, and incubated with horseradish peroxidase-conjugated secondary antibodies. The level of Nrf2, COL1A1, HO-1, and RUNX2 expression were analyzed using the Image-Pro Plus software.

### 2.19. The Level of ROS In Vivo

The level of ROS in femurs was detected using fluorescent dye DHE as previously described [27]. Briefly, femurs bones preserved in 4% paraformaldehyde (PFA) for four weeks were delicately decalcified in 10% EDTA solutions. The fixed bone tissues were cryoprotected through overnight incubation at 4°C with 20% sucrose and 2% polyvinylpyrrolidone solution. The bones were then immersed in optimum cutting temperature solution (O.C.T.) and stored at -80°C till further analyses. The bone tissues were cut into thin 5 m sections and incubated with DAPI solution and with an antifluorescence quencher (Yeasen Biotech Co., Ltd., Shanghai, China). The DHE images of the newly frozen segments were observed using a fluorescent microscope (Olympus Life Science; Tokyo, Japan).

### 2.20. TUNEL Assay

The TUNEL staining assay was performed using Cell Death Detection Kit (Yeasen Biotech Co., Ltd., Shanghai, China), following the manufacturer's instructions. Briefly, the tissues were incubated at 37°C in a humid environment with a mixture of TUNEL solution hydrolyzed with 15 g/mL proteinase K, and thereafter treated with 3% hydrogen peroxide. The percentage of apoptosis was then determined.

### 2.21. Data Analysis

Continuous normally distributed data were expressed as a mean ± SEM. All experiments were performed at least 3 times. Data were analyzed using the SPSS software (IBM, Armonk, NY, USA). Differences between groups were analyzed using the Tukey test and one-way ANOVA. Statistical significance was set at *p* value of < 0.05 (*p* < 0.05).

## 3. Results

### 3.1. HO-1 Expression and CO Content in the Femur

**Figure 1:**
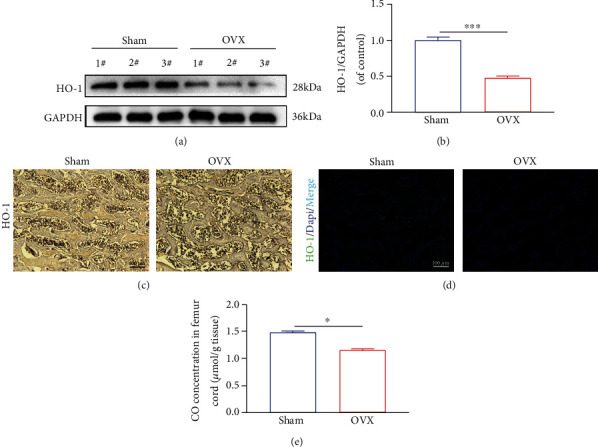
HO-1 expression and CO content in tissues. (a, b) The protein expression of HO-1 in tissues among different groups. (c) Representative immunohistochemical images of HO-1 among different groups. (d) Representative immunofluorescence staining images of HO-1 (green) and Dapi (blue) among different groups. (e) CO concentration in tissues among different groups. All experiments were repeated at least 3 times independently. Significant differences between groups are indicated as ^∗∗∗^*p* < 0.001, ^∗^*p* < 0.05.

We detected the expression of HO-1 in the distal femur, the most pivotal enzyme of autogenous CO generation. Western blotting results revealed that HO-1 expression level decreased after OVX (Figures 1(a) and 1(b)). Further, IHC and IF staining revealed that HO-1 expression decreased in the distal femur of OVX rats compared to the Sham group (Figures 1(c) and 1(d)). To ascertain the change in CO level after OVX, we quantified the CO content in different groups. We found that the CO concentration was reduced in the distal femur of the OVX rat (Figure 1(e)). These results indicated that HO-1 expression and endogenous CO were decreased after OVX.

### 3.2. Effects of CORM-3 on the Proliferation of BMSCs

**Figure 2:**
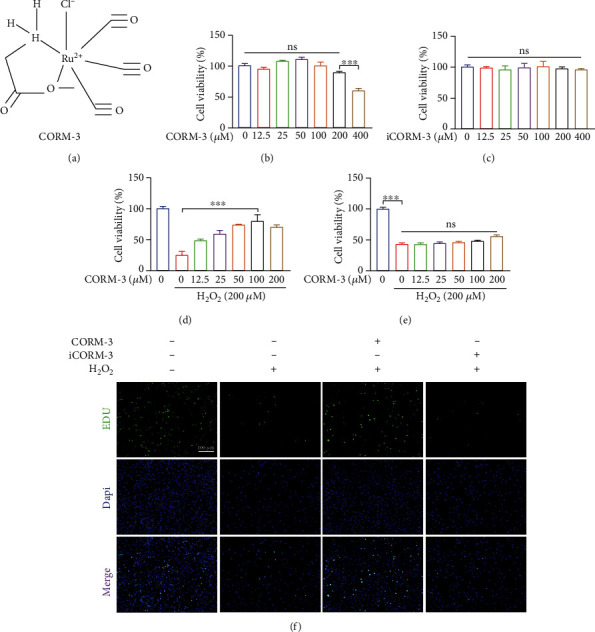
Effects of CORM-3 on BMSCs viability. (a) The chemical structure of CORM-3. (b) Percentage of viable cells receiving treatment with different CORM-3 doses for 24 hours. (c) Percentage of viable cells receiving treatment with different iCORM-3 doses for 24 hours. (d) Percentage of viable cells pretreated with H_2_O_2_ with/without different concentrations of CORM-3. (e) Percentage of viable cells pretreated with H_2_O_2_ with/without different concentrations of iCORM-3. (f) Representative fluorescent images of BMSCs labeled with EDU (green), and nuclei fluorescence was visualized with Hochest 3342 staining (blue). The data in the figures represent the averages ± SEM of 3 times in duplicates. Significant differences between groups are indicated as ^∗∗∗^*p* < 0.001; ns: no significance.

The chemical molecular formula of CORM-3 is shown in Figure 2(a). BMSCs were treated with varying CORM-3/iCORM-3 concentrations. We found that 100 *μ*m was the highest nontoxic concentration of CORM-3 to BMSCs and iCORM-3 had little impact on cell activity (Figures 2(b) and 2(c)). CORM-3 inhibited the effect of H_2_O_2_ on the viability of BMSCs in a dose-dependent manner (Figure 2(d)). However, BMSCs were also treated with varying iCORM-3 concentrations and iCORM-3 had no significant effect on the activity of BMSCs (Figure 2(e)). As already stated, 100 *μ*m of CORM-3 and 100 *μ*m of iCORM-3 were used in all subsequent vitro experiments. Meanwhile, 200 *μ*M H_2_O_2_ was used to induce intracellular oxidative stress in BMSCs. Further, the protective effect of CORM-3 on H_2_O_2_-induced BMSCs damage was assessed using the EDU incorporation assay. We found that CORM-3 increased the proliferation of BMSCs under H_2_O_2_ treatment. However, iCORM-3 did not affect the effect of H_2_O_2_ on the proliferation of BMSCs (Figure 2(f)).

### 3.3. CORM-3 Modulated the H_2_O_2_-Induced Apoptosis of BMSCs

**Figure 3:**
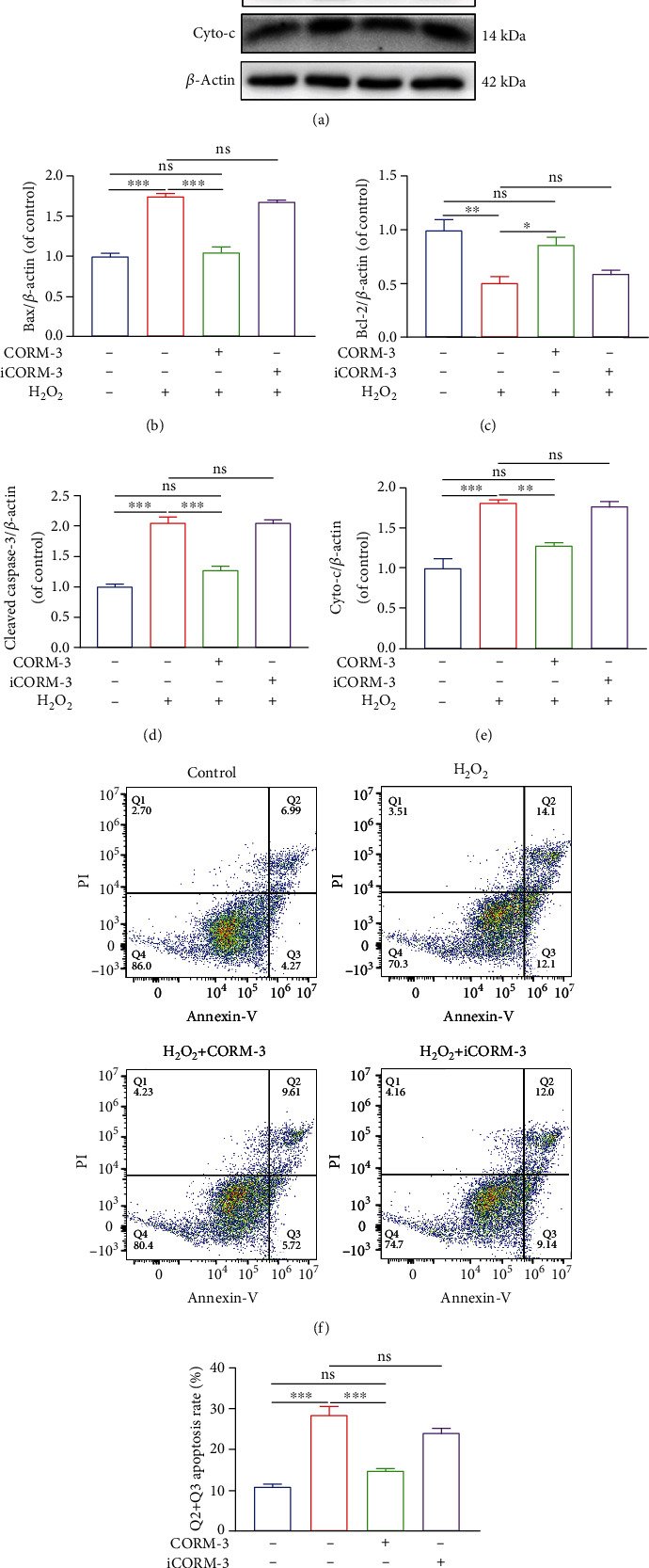
CORM-3 suppressed H_2_O_2_-induced apoptosis in BMSCs. (a–e) Expression levels of Bax, Bcl-2, cleaved caspase-3, and Cyto-C in the differentially treated BMSCs. (f, g) Representative images and quantification of cell apoptosis by flow cytometry. The data in the figures represent the averages ± SEM of 3 times in duplicates. Significant differences between groups are indicated as ^∗∗∗^*p* < 0.001, ^∗∗^*p* < 0.01, and ^∗^*p* < 0.05; ns: no significance.

In H_2_O_2_-induced cells, the expressions of proapoptotic Bax and cleaved capase-3 were upregulated. Similarly, Cytochrome C (Cyto-c), as previously reported, is upregulated early during apoptosis while antiapoptotic Bcl-2 was downregulated [28]. Notably, the levels of these proteins returned to normal levels upon CORM-3 treatment (Figures 3(a)–3(e)). The percentage of early apoptotic plus late apoptotic cells was calculated following flow cytometric analysis (Figures 3(f) and 3(g)). Considering these results, we demonstrated that CORM-3 ameliorated H_2_O_2_-induced damage on BMSCs but iCORM-3 had no effect on the H_2_O_2_-induced damage on BMSCs.

### 3.4. CORM-3 Modulates H_2_O_2_-Induced Oxidative Stress in BMSCs

**Figure 4:**
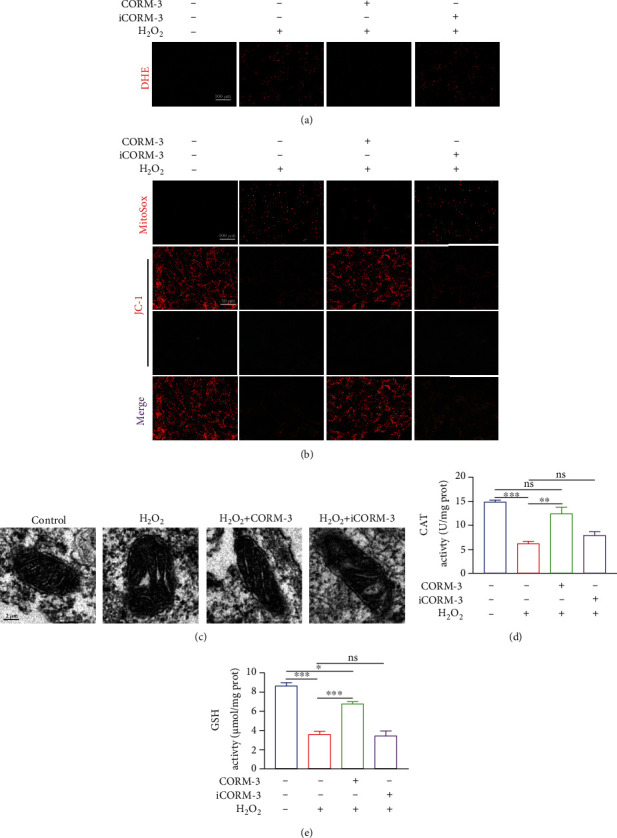
CORM-3 neutralized H_2_O_2_-induced oxidative stress and mitochondrial dysfunction in BMSCs. (a) Oxidative stress levels in treated BMSCs were detected by DHE. (b) Representative images of MitoSox and JC-1 intensities in the differentially treated BMSCs. (c) Representative images of mitochondrial morphology in the differentially treated BMSCs. (d, e) The activities of CAT and GSH in H_2_O_2_-induced cells with/without CORM-3. The data in the figures represent the averages ± SEM of 3 times in duplicates. Significant differences between groups are indicated as ^∗∗∗^*p* < 0.001, ^∗∗^*p* < 0.01, and ^∗^*p* < 0.05; ns: no significance.

Oxidative stress disrupts the normal functioning of cells. Therefore, this study assessed the impact of CORM-3 on ROS production, antioxidant enzyme activity, and mitochondrial dysfunction in H_2_O_2_-treated BMSCs. Further, DHE staining was performed to determine ROS levels in cells, whereas MitoSox and JC-1 probes were utilized to assess mitochondrial ROS and mitochondrial membrane potential (MMP), respectively. H_2_O_2_ exposure enhanced ROS production relative to untreated cells, which was neutralized by pretreatment with CORM-3 (Figure 4(a)). Compared to untreated cells, H_2_O_2_-treated cells had significantly reduced MMP and higher superoxide anion content and both recovered to near-physiological levels by CORM-3 treatment (Figure 4(b)). TEM analyses supported this finding, and we observed mitochondrial morphology deterioration following H_2_O_2_ exposure, including shrunken mitochondria, ruptured membranes, and mitochondrial recovery after CORM-3 treatment (Figure 4(c)). Coculture of H_2_O_2_-treated BMSCs with CORM-3 restored CAT and GSH-PX activities (Figures 4(d) and 4(e)). In the iCORM-3 group, the results of the above tests were similar to the H_2_O_2_-treated group.

### 3.5. CORM-3 Restored the Differentiation and Mineralization of H_2_O_2_-Treated BMSCs

**Figure 5:**
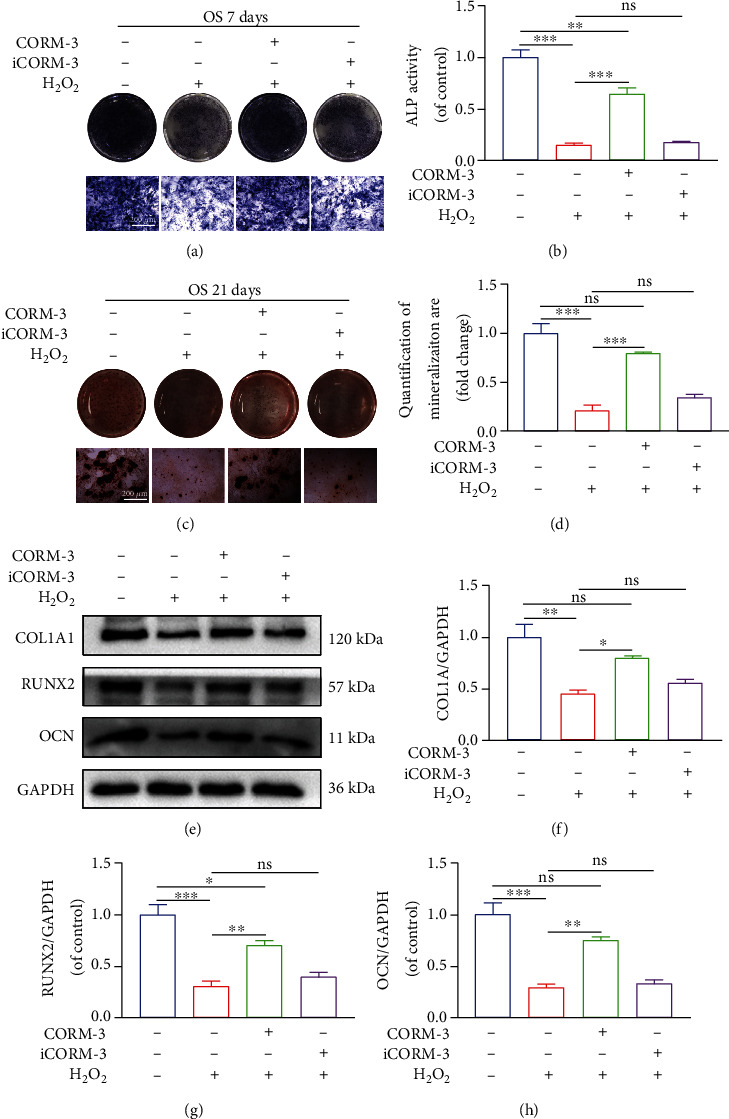
CORM-3 restored differentiation and mineralization of H_2_O_2_-induced BMSCs. (a–d) Representative images of ALP staining on the 7^th^ day and ARS staining on the 21^st^ day of osteogenic induction in differentially treated BMSCs. (e–h) Expression levels of COL1A1, RUNX2, and OCN in the differentially treated BMSCs. Data are the average ± SEM of 3 independent experiments. Significant differences between groups are indicated as ^∗∗∗^*p* < 0.001, ^∗∗^*p* < 0.01, and ^∗^*p* < 0.05; ns: no significance.

Oxidative stress and mitochondrial dysfunction inhibit the osteogenic differentiation of BMSCs. We found that based on ALP activity, H_2_O_2_ treatment significantly lowered osteogenic differentiation of BMSCs and dramatically lowered the production of calcium nodules by day 21 of treatment. However, CORM-3 treatment restored the ALP activity and mineralization of BMSCs (Figures 5(a)–5(d)), all of which upregulated the expression of COL1A1, RUNX2, and OCN 7 days after osteogenic induction (Figures 5(e)–5(h)). Overall, CORM-3 protected against the inhibitory effect of H_2_O_2_ on the differentiation and mineralization of BMSCs. However, iCORM-3 did not ameliorate the effect of H_2_O_2_ on BMSCs.

### 3.6. CORM-3 Exerted Its Protective Effect against H_2_O_2_ via the Nrf2/HO-1 Signaling Pathway

**Figure 6:**
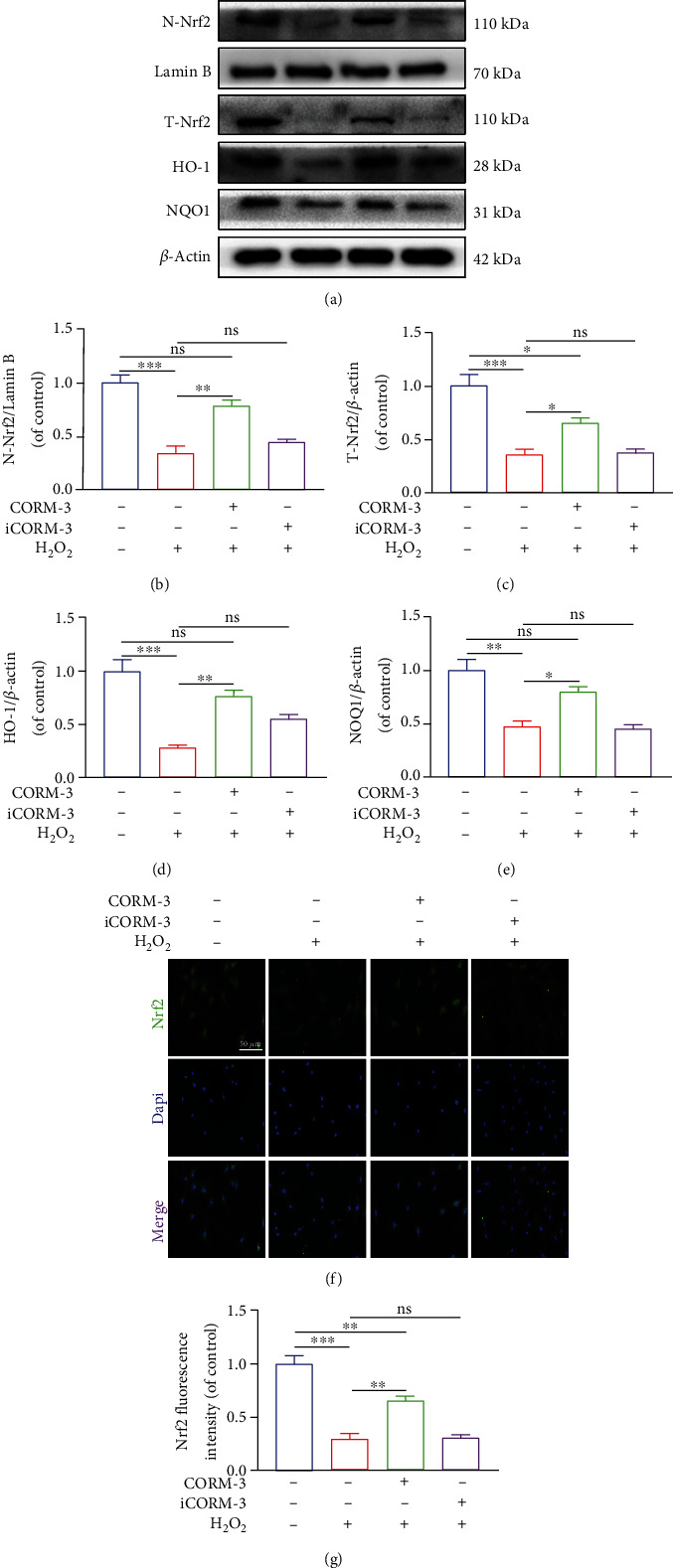
CORM-3 activated the Nrf2 pathway in BMSCs. (a–e) Immunoblot of Nrf2, NQO1, and HO-1 levels in the differentially treated BMSCs. (f, g) Representative fluorescence images of Nrf2 localization in the differentially treated cells. Data are the average ± SEM of 3 independent experiments. Significant differences between groups are indicated as ^∗∗∗^*p* < 0.001, ^∗∗^*p* < 0.01, and ^∗^*p* < 0.05; ns: no significance.

**Figure 7:**
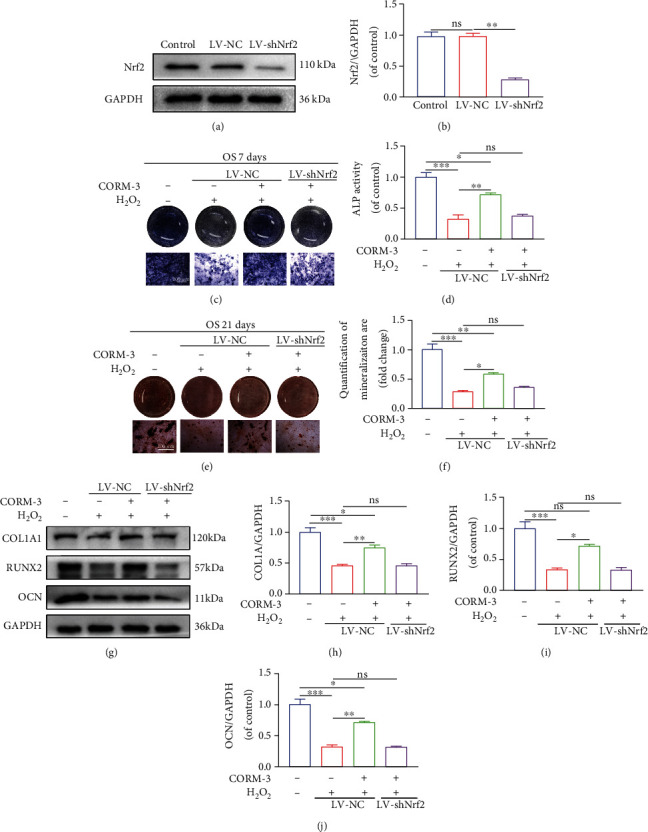
The Nrf2 pathway mediated the osteoprotective effects of CORM-3. (a, b) Western blotting was performed to assess the knockdown of Nrf2 after LV-shNrf2 transfection. (c–f) Representative images of ALP staining on the 7^th^ day and ARS staining on the 21^st^ day of osteogenic induction in differentially treated BMSCs. (g–j) Immunoblot of COL1A1, RUNX2, and OCN in the differentially treated BMSCs. Data are the average ± SEM of 3 independent experiments. Significant differences between groups are indicated as ^∗∗∗^*p* < 0.001, ^∗∗^*p* < 0.01, and ^∗^*p* < 0.05; ns: no significance.

CORM-3 stimulates the Nrf2/HO-1 signaling pathway in an ischemia-reperfusion model of myocardium cell injury, suggesting that the protective effects of CORM-3 are mediated through this pathway. NAD(P)H quinone oxidoreductase 1 (NQO1) is an essential homodimer flavin protease that is against endogenous oxidative stress by conserving the reduced form of ubiquinone and tocopherol. CORM-3 treatment reversed the H_2_O_2_ inhibitory effects on Nrf2 nuclear translocation and expression of HO-1 and NQO1 (Figures 6(a)–6(g)). Interestingly, suppressing Nrf2 expression inhibited the protective effect of CORM-3 against H_2_O_2_. Western Blot shows that Nrf2 was knocked out successfully (Figures 7(a) and 7(b)). Seven days after osteogenic induction, the level of expression of COL1A1, RUNX2, and OCN in BMSCs treated with CORM-3 was partially reduced by Nrf2 knockout (Figures 7(g)–7(j)) and reversed CORM-3's beneficial impacts on BMSCs' mineralization and differentiation (Figures 7(c)–7(f)). These findings demonstrated that CORM-3 exerts its effects by inducing nuclear translocation of Nrf2 and activating its downstream pathways.

### 3.7. CORM-3 Inhibited Osteoclastogenesis and M1/M2 Polarization

**Figure 8:**
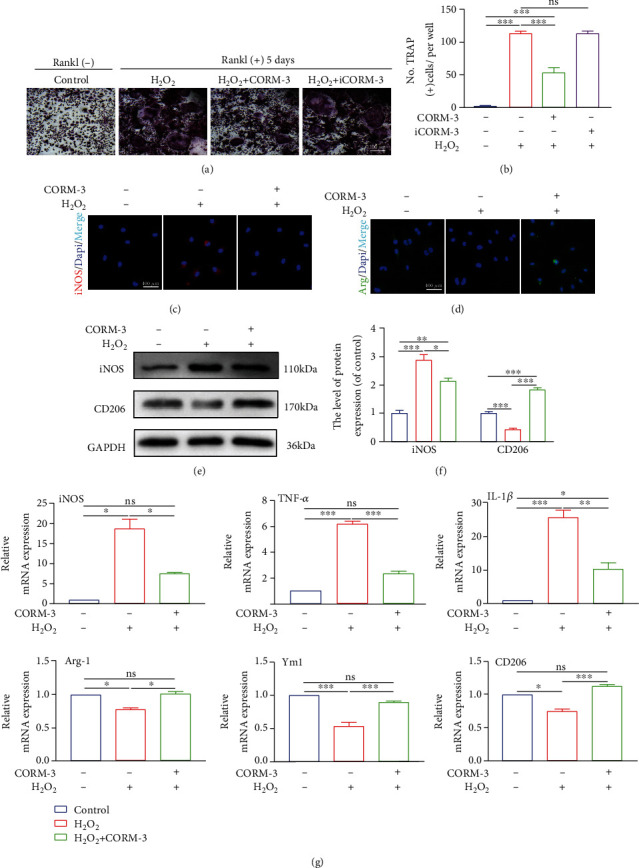
CORM-3 modulated M1 subtype macrophage polarization and inhibited osteoclast formation. (a, b) Representative images of TRAP staining of BMMs cells cultured with different treatments in the presence of RANKL and M-CSF for 5 days. (c, d) Representative images of immunofluorescence staining about inducible iNOS, CD206, and DAPI in BMMs among different groups. (e, f) Relative expression of M1 and M2 markers such as iNOS and CD206 at the protein level. (g) Relative expression of M1 and M2 markers such as iNOS, TNF-a, IL-1*β*, Arg-1, Ym1, and CD206 at mRNA level. Data are the average ± SEM of 3 independent experiments. Significant differences between groups are indicated as ^∗∗∗^*p* < 0.001, ^∗∗^*p* < 0.01, and ^∗^*p* < 0.05; ns: no significance.

Numerous cytokines and microbial metabolites modulate macrophage polarization. Macrophages can be polarized into two different phenotypes. H_2_O_2_ induces polarization of macrophages into the M1 phenotype and upregulates the expression of TNF-*α*, iNOS, and IL-1*β*. Contrarily, it suppresses the polarization of the M2 macrophages but increases the expression of Arg-1, Ym-1, and CD206. Modulating the M1/M2 polarization of macrophages may be a new approach to treating osteoporosis. Therefore, we speculated that CORM-3 reduces inflammation and inhibits osteoclastosis by decreasing the M1/M2 ratio. Immunofluorescence analysis revealed that H_2_O_2_ treatment significantly increased the proportion of iNOS-positive cells, but CORM-3 treatment reversed this phenomenon (Figure 8(c)). Also, CORM-3 treatment significantly increased the proportion of the CD206-positive cells (Figure 8(d)). In addition, the CORM-3 treatment modulated the mRNA expression for iNOS, TNF-*α*, IL-1*β*, Arg-1, Ym1, and CD206 (Figure 8(g)). The expression of iNOS and CD206 proteins exhibited a similar trend (Figures 8(e)–8(f)). Further analyses revealed that CORM-3 decreases the proportion of TRAP positive cells (Figures 8(a) and 8(b)). These findings suggested that CORM-3 promotes the polarization of macrophages from M1 to M2 phenotypes and inhibits osteoclastosis.

### 3.8. CORM-3 Treatment Restored Bone Microstructure and Promoted Bone Synthesis In Vivo

**Figure 9:**
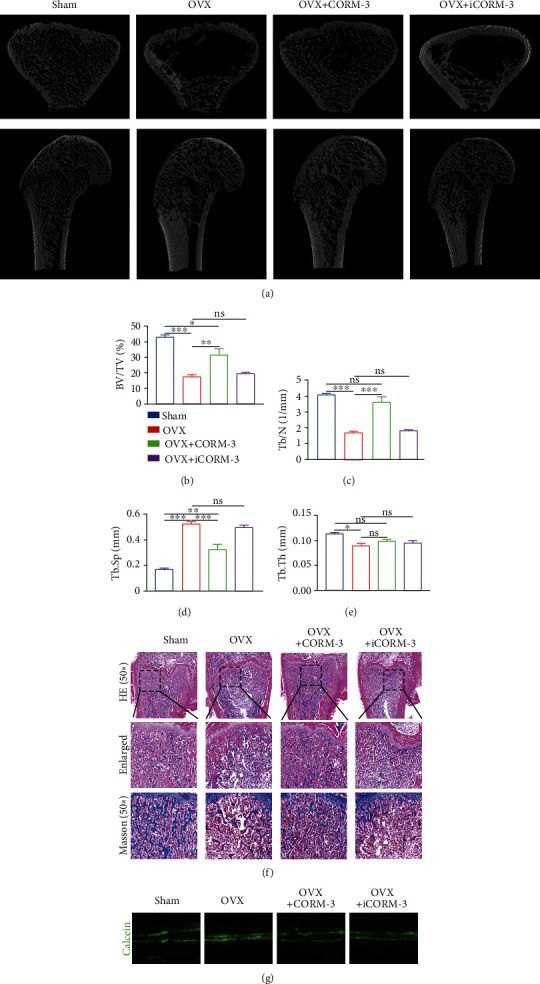
CORM-3 treatment revived the microstructure and augmented bone formation of the rats. (a–e) Representative micro-CT images of the longitudinal and transverse sections of the distal femurs and the BV/TV, Tb.N, Tb.Sp, Tb.Th values in the differentially treated animals. (f) Representative images of H&E staining and Masson's staining of the metaphyseal tissue sections of the thigh. (g) Representative images of calcein double-labeling of the distal femur. Data are expressed as the average ± SEM of 3 times in duplicates. Significant differences between groups are indicated as ^∗∗∗^*p* < 0.001, ^∗∗^*p* < 0.01, and ^∗^*p* < 0.05; ns: no significance.

The OVX rat model was used to evaluate the effect of CORM-3 on osteoporosis in vivo. Analysis of 3-D micro-CT scan images revealed that 8 weeks of CORM-3 treatment restored the architecture of trabecular bone in distal femurs (Figure 9(a)). Additionally, OVX rats were administered with CORM-3 for 8 weeks also showed significant improvements in BV/TV, Tb.N, and Tb.Sp (Figures 9(b)–9(d)). However, no significant change in the Tb.Th was observed (Figure 9(e)). Consistently, H&E and Masson trichrome staining of the distal femur sections indicated more numerous trabeculae and distinct lower trabecular separation in the OVX+CORM-3 group compared to OVX and OVX+iCORM-3 group (Figure 9(f)). The effect of CORM-3 on bone formation in OVX rats was analyzed using calcein double labels. calcein was injected twice (on days 2 and 14) before sample extraction and analysis. We found that compared to control, the interlabel distance was significantly increased in the CORM-3 group (Figure 9(g)).

### 3.9. CORM-3 Injection Restored Nrf2 Expression and Osteogenic Indices of the Distal Femur and Reduced the Production of ROS and Tissue Apoptosis In Vivo

**Figure 10:**
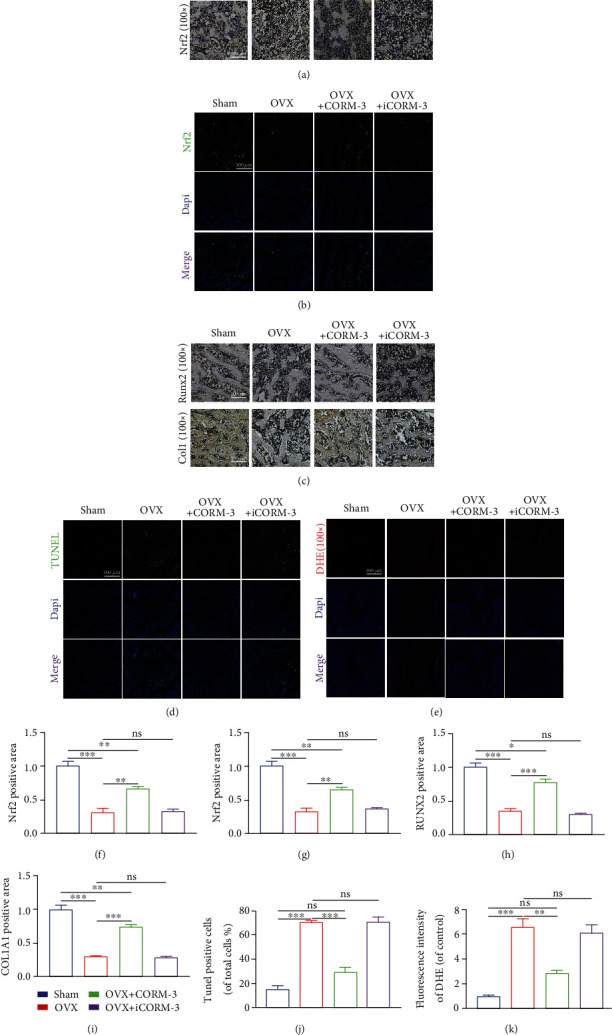
CORM-3 treatment increased osteogenic and Nrf2 expression, suppressed apoptosis, and attenuated ROS level in vivo. (a, f) Nrf2 immunohistochemical staining image of the metaphyseal tissue of the thigh. (b, g) Nrf2 immunofluorescence image of the metaphyseal tissue of the thigh. (c, h, i) COL1A1 and RUNX2 immunohistochemical staining. (d, j) Representative images and quantification of apoptosis-positive cells in distal femora were determined by TUNEL assay. (e, k) Representative DHE images of the distal femoral and fluorescence quantification of different groups. Data are expressed as the average ± SEM of 3 times in duplicates. Significant differences between groups are indicated as ^∗∗∗^*p* < 0.001, ^∗∗^*p* < 0.01, and ^∗^*p* < 0.05; ns: no significance.

To test whether Nrf2 has the same trend in rats, we determined the level of Nrf2 expression in rats' distal femurs using IHC and IF. Nrf2 levels were significantly elevated in CORM-3-treated distal femurs compared to OVX-treated distal femurs (Figures 10(a), 10(b), 10(f), and 10(g)). Next, IHC staining was performed on the distal femur to detect osteogenesis in vivo. Similar to in vitro results, RUNX2 and COL1A1 levels were significantly higher in the CORM-3 group than in the OVX group (Figures 10(c), 10(h), and 10(i)). Furthermore, TUNEL assays were utilized to determine the level of apoptosis in the distal femurs of the various treatment groups. The results reveal that the OVX group had a significantly greater index of apoptotic cells in the distal femur than the sham group. The TUNEL-positive cell percentage was also considerably lowered in the CORM-3 treatment group (Figures 10(d) and 10(j)). Finally, the femur ROS level was evaluated by DHE staining. DHE staining revealed that COMR-3 modulated the oxidative stress in the distal femur induced by H_2_O_2_ treatment (Figures 10(e) and 10(k)).

## 4. Discussion

The correlations between osteoporosis and ROS and underlying molecular mechanism have generated tremendous interest over the years. Skulachev et al. found high ROS levels in almost all osteoporosis forms, including senile osteoporosis, menopausal osteoporosis and diabetic osteoporosis, suggesting that ROS independently promotes osteoporosis [29, 30]. As reported, ROS can be endogenously eliminated by the HO-1/CO pathway [31]. Up to date, little is known regarding the effect of CO in OVX rats. To the best of our knowledge, we are the first to report the relationship between HO-1 and its product CO in OVX. We found that HO-1 and its product CO significantly decreased in the OVX rat model compared to the controls. In addition, our experiments showed that supplementing exogenous CO reversed H_2_O_2_-induced mitochondrial dysfunction by activating the Nrf2/HO-1 signaling pathway and inhibited osteoclast differentiation by regulating the macrophage polarization and, thus, preventing osteoporosis. Given that iCORM-3 does not exert the same effects as CORM-3, directly implicating CO as the active mediator in these responses.

As one of the degradation products of heme, CO possesses diverse biological roles [14]. However, because of its high affinity to Hb, CO is poisonous to living animals [31]. CORM-3 is a novel compound that carriers of CO and reproduce its biological actions [32]. Numerous studies have demonstrated that CORM-3 is non-toxic in low doses. For instance, Liu et al. found that CORM-3 ameliorates acute pancreatitis, and Portal et al. reported that CORM-3 protects adult cardiomyocytes against hypoxia-reoxygenation by modulating pH restoration [33, 34]. Accordingly, we speculated that CORM-3 plays a vital role in the metabolism of bone. CORM-3 inhibits osteoclastogenic differentiation by the HO-1 pathway [35]. Similarly, Li et al. found that CORM-3 promotes the osteogenic differentiation of BMSCs [19]. Therefore, we performed animal experiments to verify the therapeutic effect of CORM-3 in vivo. We found that CORM-3 ameliorated bone loss in OVX rats in vivo.

The mitochondrion is a major site for ROS production, and excessive ROS accumulation disrupts the mitochondrial membrane potential and impairs the mitochondrial function, triggering apoptosis [24]. Growing evidence shows that maintaining mitochondrial homeostasis is critical for osteogenic differentiation of BMSCs [36]. According to Chen et al.'s discovery, BMSCs with compromised mitochondria displayed lower osteogenic protein expression levels, accompanied by decreased ALP activities and reduced mineralized nodule formation [37]. Pal et al. reported that restoring the mitochondrial membrane potential promoted osteogenic differentiation [38]. In the present study, we found that CORM-3 treatment attenuated the deleterious impact of H_2_O_2_ and downregulated the expression of apoptosis-related proteins, including Cyto-C in mitochondria, Bax, and cleaved caspase-3, but upregulated that of Bcl2, an antiapoptotic protein. Furthermore, H_2_O_2_ treatment suppressed the expression of osteogenic-related proteins, including COL1A1, RUNX2, and OCN, accompanied by concomitant loss of ALP and ARS osteogenic phenotypes. However, CORM-3 treatment upregulated the expression of osteogenic phenotypes of these proteins and restored mitochondrial function and structure, consistent with previous findings [39]. ROS has previously been reported to induce macrophage polarization to the M1 phenotype, promoting osteoclastogenesis [40, 41]. Herein, qPCR, immunofluorescence, and TRAP staining confirmed that CORM-3 regulates H_2_O_2_-induced macrophage polarization and inhibits osteoclastogenesis. Taken together, we suppose that CORM-3 prevents BMSCs apoptosis due to its inhibition of mitochondrial dysfunction and inhibits osteoclastogenesis by regulating macrophage polarization.

The transcription factor Nrf2 is the primary sensor of oxidative stress to increase the downstream expression of antioxidant enzymes upon activation, including HO-1 and NQO1 [42, 43]. It has been reported to interact with antioxidant response element (ARE) leading to the expression of antioxidant enzymes [44]. Pan et al. found that CO stimulates nuclear Nrf2 accumulation, resulting in increased binding to both ARE sequences of HO-1 promoter and leading to HO-1 expression in RAB-1 cells [35, 45]. Our results indicated that HO-1 expression and endogenous CO was decreased in distal femur after OVX. Meanwhile, exogenous CO supplementation upregulates Nrf2/HO-1 pathway. Another previous study reported that CORM-3 activates Nrf2 expression in hepatocellular cancer cells. It is consistent with our results that CO supplementation induces the expression of the antioxidant proteins HO-1 and the antioxidant enzyme NQO1 and removes ROS by promoting the nuclear translocation of Nrf2 [46]. In the present study, we found that knocking off Nrf2 expression significantly weakened the therapeutic effect of CORM-3. These results also confirmed previous findings that CORM-3 supplementation exerts its antioxidant properties through the Nrf2/HO-1 signaling pathway [47]. Finally, the OVX model showed that in vivo, injection of CORM-3 increased the expression of osteogenic-related indicators in the distal femur, reduced ROS accumulation in the femur, reduced bone loss, and upregulated Nrf2 expression in the distal femur. Upadhyay et al. reported that the molecular docking profile of CO in the kelch domain of Kelch-like ECH-associated protein 1 (Keap1) protein suggested that CO released mediated Nrf2 activation. Meanwhile, CO promotes Nrf2 activation via CO-mediated release of Nrf2 from Keap1 in HepG2 cells was reported by them [48, 49]. Furthermore, Wang et al. found that CO could activate different kinases such as phosphatidylinositol 3-kinase, protein kiinase C, c-Jun NH2-terminal kinase, p38, and extracellular-signal-regulated kinase, which lead to Nrf2 activation and downstream gene expression [50]. However, the detailed mechanisms between CO and Nrf2/HO-1 still need our further research. All in all, the current results demonstrate that CO released by CORM-3 upregulates HO-1 expression and Nrf2 activity to attenuate osteoporosis for the first time.

**Figure 11:**
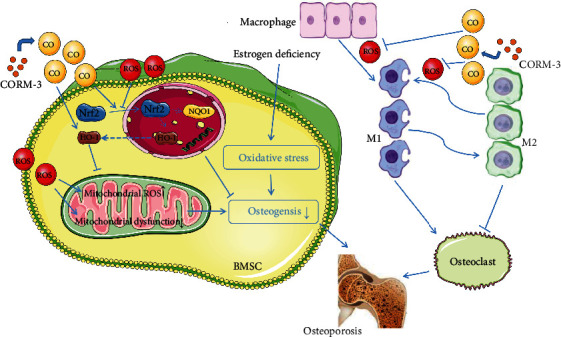
Mechanisms of CORM-3 action on BMSCs. CORM-3 reduced H_2_O_2_-induced ROS accumulation and mitochondrial dysfunction in BMSCs and regulated macrophage polarization to inhibit osteoclast and prevent bone loss by activating the Nrf2/HO-1 pathway.

In summary, these results indicated that exogenous CORM-3 protects against H_2_O_2_-induced apoptosis, oxidative stress, and mitochondrial damage in BMSCs in vitro by activating the Nrf2/HO-1 signaling pathway. Furthermore, CORM-3 promotes M1/M2 polarization of macrophages, inhibits osteoclastogenesis, and delays osteoporosis in vivo and in vitro. This study tentatively suggest that the mechanisms of action of CORM-3 are shown in Figure 11. The results of this study reveal a new theoretical basis for osteoporosis inhibition in cells.

## Data Availability

The data used to support the findings of this study are available from the corresponding author upon request.
